# Vortioxetine restores reversal learning impaired by 5-HT depletion or chronic intermittent cold stress in rats

**DOI:** 10.1017/S1461145714000571

**Published:** 2014-05-23

**Authors:** Ashley Wallace, Alan L. Pehrson, Connie Sánchez, David A. Morilak

**Affiliations:** 1 Department of Pharmacology and Center for Biomedical Neuroscience, University of Texas Health Science Center at San Antonio, San Antonio, TX 78229, USA; 2 Lundbeck Research USA, Paramus, NJ 07652, USA

**Keywords:** Antidepressant, chronic stress, cognitive flexibility, orbitofrontal cortex, reversal learning, serotonin

## Abstract

Current treatments for depression, including serotonin-specific reuptake inhibitors (SSRIs), are only partially effective, with a high incidence of residual symptoms, relapse, and treatment resistance. Loss of cognitive flexibility, a component of depression, is associated with dysregulation of the prefrontal cortex. Reversal learning, a form of cognitive flexibility, is impaired by chronic stress, a risk factor for depression, and the stress-induced impairment in reversal learning is sensitive to chronic SSRI treatment, and is mimicked by serotonin (5-HT) depletion. Vortioxetine, a novel, multimodal-acting antidepressant, is a 5-HT_3_, 5-HT_7_ and 5-HT_1D_ receptor antagonist, a 5-HT_1B_ receptor partial agonist, a 5-HT_1A_ receptor agonist, and inhibits the 5-HT transporter. Using adult male rats, we first investigated the direct effects of vortioxetine, acting at post-synaptic 5-HT receptors, on reversal learning that was compromised by 5-HT depletion using 4-chloro-DL-phenylalanine methyl ester hydrochloride (PCPA), effectively eliminating any contribution of 5-HT reuptake blockade. PCPA induced a reversal learning impairment that was alleviated by acute or sub-chronic vortioxetine administration, suggesting that post-synaptic 5-HT receptor activation contributes to the effects of vortioxetine. We then investigated the effects of chronic dietary administration of vortioxetine on reversal learning that had been compromised in intact animals exposed to chronic intermittent cold (CIC) stress, to assess vortioxetine's total pharmacological effect. CIC stress impaired reversal learning, and chronic vortioxetine administration prevented the reversal-learning deficit. Together, these results suggest that the direct effect of vortioxetine at 5-HT receptors may contribute to positive effects on cognitive flexibility deficits, and may enhance the effect of 5-HT reuptake blockade.

## Introduction

Executive functions involving the prefrontal cortex (PFC) are disrupted in patients with depression (Merriam et al., 1999; Jaeger et al., 2006; Herrera-Guzmán et al., 2010). One such executive process is cognitive flexibility, the ability to modify previously learned associations and behavioral patterns in response to a changing environment (Kehagia et al., 2010). Impairments in cognitive flexibility contribute to the perseverative emotional biases that are important in the development and persistence of depressive symptoms (Beck, 1976; Mathews and Mackintosh, 1998; Coles and Heimberg, 2002). Depressed patients exhibit cognitive biases for emotionally meaningful material, particularly related to stressful life events (Beck, 1976), a narrowing of attention to depression-relevant thoughts, and difficulty shifting cognitive set, all consistent with the perseverative focus on themes of loss and worthlessness, and the persistent ruminations that are prevalent in depression (Fossati et al., 1999; Merriam et al., 1999; Murphy et al., 1999; Austin et al., 2001). More effective management of cognitive impairment, specifically of cognitive inflexibility, may be important for more effective treatment of depression (Naismith et al., 2007). Indeed, current treatments for depression, including serotonin-specific reuptake inhibitors (SSRIs), are only partially effective, with a high incidence of residual symptoms, relapse, and treatment resistance (Fava, 2006; Jaeger et al., 2006). Clinical research suggests that only half of depressed patients respond to initial treatment with an SSRI, and only one-third eventually achieve remission (Gaynes et al., 2009). Thus, more efficacious treatments are needed.

Chronic stress is a risk factor for developing depression (Kessler, 1997; Kendler et al., 1999; Caspi et al., 2003). Moreover, dysregulation of serotonergic neurotransmission can interact with stress to increase risk for depression (Caspi et al., 2003). In previous studies using the attentional set-shifting test (AST) to measure cognitive flexibility in rats (Birrell and Brown, 2000), we have shown that serotonergic neurotransmission in the orbitofrontal cortex (OFC) modulates reversal learning, one form of cognitive flexibility measured by the AST (Lapiz-Bluhm et al., 2009; Lapiz-Bluhm and Morilak, 2010; Furr et al., 2012). Further, chronic intermittent cold (CIC) stress induces a selective deficit in reversal learning that is sensitive to chronic treatment with an SSRI (Lapiz-Bluhm et al., 2009; Lapiz-Bluhm and Morilak, 2010).

Vortioxetine is a novel antidepressant with multimodal action approved by the FDA for the treatment of major depressive disorder (FDA, 2013). In addition to blocking the serotonin (5-HT) transporter, vortioxetine is an antagonist at 5-HT_3A_, 5-HT_7_ and 5-HT_1D_ receptors, a partial agonist at 5-HT_1B_ receptors, and a full agonist at 5-HT_1A_ receptors (Bang-Anderson et al., 2011; Westrich et al., 2012). Preclinical and clinical studies have demonstrated antidepressant properties of vortioxetine (Alvarez et al., 2012; Katona et al., 2012; Mørk et al., 2012), yet little is known about the relative contributions of 5-HT reuptake inhibition and direct receptor mechanisms to the beneficial behavioral effects of vortioxetine. In the first part of this study, we investigated the direct effects of vortioxetine at post-synaptic 5-HT receptors on reversal learning that was compromised by 5-HT depletion using 4-chloro-DL-phenylalanine methyl ester hydrochloride (PCPA), a tryptophan hydroxylase inhibitor, effectively eliminating any contribution of 5-HT reuptake inhibition. In the second part of the study, we investigated the effects of chronic vortioxetine administration on reversal learning in intact animals compromised by exposure to CIC stress (Lapiz-Bluhm et al., 2009; Lapiz-Bluhm and Morilak, 2010), thus allowing the full spectrum of vortioxetine's pharmacological mechanisms to be exerted.

## Methods

### Animals

A total of 113 adult male Sprague Dawley rats (Harlan Laboratories, USA) were used. Rats weighed 220–240 g upon arrival in Experiment 1 and 200–220 g upon arrival in Experiment 2. Rats were given 1 wk to acclimatize, then individually housed on a 12 h light/dark cycle (lights on 07:00 hours) with food and water available *ad libitum* until 10 d prior to testing in the AST. Experiments were conducted during the light phase of the cycle. All procedures were reviewed and approved by the Institutional Animal Care and Use Committee of the UTHSCSA, and were consistent with NIH guidelines for the care and use of laboratory animals. All efforts were made to minimize pain, distress, and the number of animals used.

### Attentional set-shifting test (AST)

An abbreviated AST was conducted according to published procedures (Lapiz-Bluhm and Morilak, 2010), but only through completion of the first reversal task, which we have shown previously to be compromised selectively by both 5-HT depletion and CIC stress (Lapiz-Bluhm et al., 2009). 10 d prior to testing, rats were placed on a restricted diet of 14 g/day of food, with water freely available. The testing apparatus was a rectangular white wooden arena with a removable divider separating one-third the length of the arena into a start box and holding area. To begin each trial, the rat was placed in the start box and given access to the rest of the arena by raising the divider. A white Plexiglas panel divided the far third of the arena into two sections. During testing, a small terracotta pot was placed in each section, and each pot was defined by a pair of cues along two stimulus dimensions; the digging medium with which it was filled, and an odor applied to the inner rim. One-quarter of a Honey Nut Cheerio (General Mills Cereals, USA) was buried 2 cm below the surface of the digging medium in the ‘positive’ pot. In all discrimination trials, a small quantity of powdered Cheerio was sprinkled onto the medium in both pots to ensure that the rat learned the discrimination and was not making choices by smelling the reward. The behavioral procedure was conducted over 3 d:

#### Day 1 – habituation

Two unscented pots were placed in the home cage and re-baited every 5 min, covering the Cheerio with increasing amounts of bedding (three trials with no bedding, three with the pots one-third full, three half-full and three completely full). The rat was then transferred to the testing arena and given three consecutive trials to retrieve the reward from both pots filled with bedding.

#### Day 2 – training

Rats were trained on two simple discriminations, to a criterion of six consecutive correct responses in each. In the first, both pots were filled with the same medium (bedding) and scented with different odors (lemon *vs.* rosewood), with only one odor associated with reward. After reaching the criterion, two unscented pots were used, each filled with a different medium (shredded paper *vs.* felt strips). All rats were trained using the same stimuli in the same order. The positive and negative cues for each rat were randomly determined. These training stimuli were not used during testing.

#### Day 3 – testing

Rats were tested on a series of three discriminations. To proceed to the next, they had to reach criterion of six consecutive correct trials. The first was a simple discrimination (SD), similar to the training trials, involving only one stimulus dimension. Half the rats were required to discriminate between two odors, only one of which was associated with reward, with both pots filled with sawdust. The other half were required to discriminate different digging media, with both pots unscented (for clarity, the remainder of this description will refer to the example with odor discrimination). The second stage was a compound discrimination (CD), in which the same discrimination was required (e.g. odor), but the second, irrelevant stimulus was introduced. Only one odor was associated with reward, and the two digging media were paired randomly with the odors over successive trials. The final stage was the reversal task, in which the same odors and media were used, and odor was still the relevant dimension, but the negative odor from the previous stage was now positive, and the positive odor from the previous stage was now negative. The same was true for rats tested with medium as the initial discrimination. The dependent measure was number of trials to reach criterion (TTC) of 6 consecutive correct responses on the reversal task.

### Experiment 1: effects of acute and sub-chronic vortioxetine treatment on reversal learning impairment induced by 5-HT depletion

The tryptophan hydroxylase inhibitor, PCPA (4-chloro-DL-phenylalanine methyl ester hydrochloride; Sigma) was prepared fresh daily by dissolving in 0.9% saline (78 mg/ml of the PCPA-salt), adjusted to pH 6.0 with 0.1 m NaOH. Thirty-eight rats were randomly assigned to two groups, pretreated for four consecutive days (days 1–4) with an injection of either vehicle (3 ml/kg, i.p.) or PCPA (200 mg/kg, calculated as the free base). This treatment has previously produced 94% depletion of 5-HT, and a reversal learning impairment (Lapiz-Bluhm et al., 2009).

**Fig. 1.**
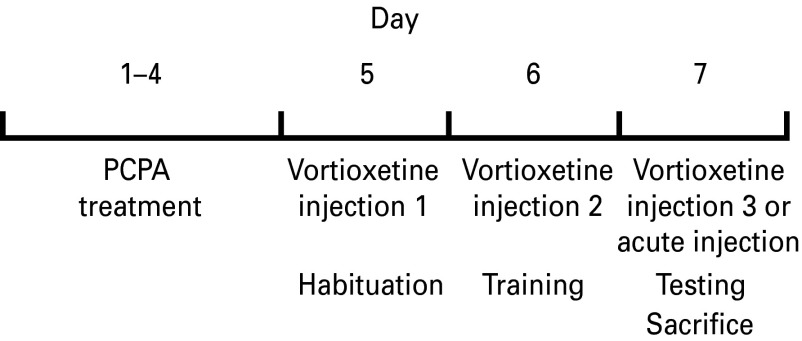
Schematic diagram and timeline for the procedures in experiment 1.

Vortioxetine (1-[2-(2,4-dimethylphenyl-sulfanyl)-phenyl]-piperazine) DL-lactate (H. Lundbeck A/S, Denmark) was prepared daily by dissolving with sonication in 10% aqueous hydroxypropyl-ß-cyclodextrin (HBC) in filtered saline. On days 5–7, corresponding to the habituation, training and testing days on the AST, two subsets of rats from each pretreatment condition were injected with either HBC vehicle (3 ml/kg, i.p.) or vortioxetine (10 mg/kg of the base) once per day, 30 min prior to the behavioral procedure for that day (see schematic Fig. 1). In addition, to test the effect of a single acute vortioxetine administration given immediately prior to testing, an additional group of PCPA-treated rats (*n* = 8) were habituated and trained on days 5–6, with no drug treatment on these days. On day 7, a single injection of vortioxetine was then administered 30 min prior to testing on the AST.

After behavioral testing, rats were sacrificed by rapid decapitation. The brain was placed in a chilled brain matrix, and a 2 mm coronal slab cut from 2–4 mm caudal to the frontal pole. A wedge of tissue containing the OFC was then dissected on ice. First, a horizontal cut was made from the rhinal sulcus to the midline. A second cut was made from the lateral surface of the brain, tangential to the ventral boundary of the forceps minor, intercepting the horizontal cut at the medial edge of the forceps minor. Tissue was flash frozen and stored at −80°C until 5-HT concentration was measured by HPLC with tandem mass spectrometry (LC/MS/MS).

### Analysis of 5-HT concentration in OFC tissue homogenates

Tissue samples from the OFC (7–40 mg wet weight) were mixed 1:4 (w/v) with 0.2% acetic acid/water and homogenized at 8°C for 4 min in a Covaris sonicator using 100 *μ*l sample tubes. [^2^H_4_]-5-HT was used as internal standard. Homogenates were transferred into Vivaspin 2 filters (2 kDa cutoff; Sartorius) and centrifuged at 4°C for 1 h at 13 500 ***g***. The supernatant was transferred into CMA tubes for LC/MS/MS analysis of 5-HT. A Waters Acquity HPLC system equipped with an YMC ODS AQ 2 × 100 mm, 3 *μ*m particle column isolated 5-HT prior to detection by a Waters Quattro Premier XE triple quadrupole mass spectrometer in MS/MS mode. Column and pre-column tubing were maintained at 40°C during elution of analytes with a mobile phase consisting of aqueous component A (0.5% formic acid in milliQ water) and organic component B (1% formic acid in acetonitrile). Gradient elution included 2 min hold at 100% A followed by a shallow gradient of 0–30% B. Run time was 9 min. Detection limit was 1 ng/ml 5-HT. Samples were analyzed in triplicate where volume permitted.

### Experiment 2: effects of chronic dietary administration of vortioxetine on reversal learning compromised by chronic intermittent cold (CIC) stress

**Fig. 2.**
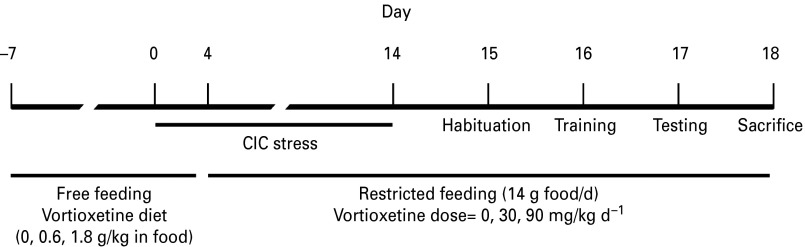
Schematic diagram and timeline for the procedures in experiment 2.

Fifty-four rats were randomly assigned to three groups fed for 25 d on a diet of control chow (Purina 5001 Rodent Chow), chow containing 0.6 g/kg vortioxetine, or chow containing 1.8 g/kg vortioxetine (prepared by Research Diets, Inc.). These preparations were calculated to deliver daily doses of approximately 0, 30 or 90 mg/kg vortioxetine during the 10 d period of food restriction (14 g/day) before testing on the AST, based on an estimated average body weight of 280 g during this period. These doses were chosen to achieve pharmacologically relevant target occupancies (Pehrson, unpublished data). After 1 wk free-feeding on the assigned diet, subsets of rats in each group were assigned to either control or CIC stress treatments (see schematic Fig. 2).

### Chronic intermittent cold (CIC) stress

CIC stress was conducted as described previously (Lapiz-Bluhm and Morilak, 2010). During the light phase of the cycle, rats were weighed and transported in their home cages with food, water, and bedding into a cold room at 4°C for 6 h, then returned to housing. This was repeated daily for 14 d. Unstressed controls were weighed and returned to housing.

As described above, food was restricted to 14 g/d for 10 d prior to testing on the AST, i.e. during the last 7 d of stress plus 2 d preceding behavioral testing. Beginning the day after the final stress treatment, rats were habituated, trained and tested on day 3. After testing was complete, they were given 14 g of the designated diet to maintain drug dosing on the test day and were sacrificed the following day. Brains were flash frozen and stored at −80°C. Fractional occupancies at the 5-HT transporter and 5-HT_1B_ receptor were measured in each dietary treatment group using *ex vivo* autoradiography.

### Estimation of fractional target occupancies via *ex vivo* autoradiography

Fractional target occupancies at the 5-HT transporter and 5-HT_1B_ receptor were estimated as described previously (Pehrson et al., 2013). Coronal brain sections were cut at 20 *μ*m, beginning approximately 1.2 mm anterior to bregma (Paxinos and Watson, 1998). Three sections from each brain were mounted on slides and stored at −20°C. For 5-HT transporter occupancy, slides were thawed and incubated for 60 min in assay buffer (50 mm Tris HCl, 150 mm NaCl, 5 mm KCl, pH 7.4) containing 4.5 nm [^3^H]-escitalopram. Non-specific binding was determined by including 1 *μ*m paroxetine. For 5-HT_1B_-receptor occupancy, slides were pre-incubated for 3 min at 4°C in assay buffer (170 mm Tris HCl, 4 mm CaCl_2_, 0.1% L-ascorbic acid, pH 7.4). After air-drying for 45 min, slides were incubated in assay buffer containing 1 nm of the 5-HT_1B_-receptor antagonist [^3^H]GR125743 and 10 *μ*m pargyline. Non-specific binding was determined using 10 *μ*m SB216641. For both assays, slides were rinsed 2 × 5 min in cold assay buffer, air dried for 30 min and transferred to a vacuum desiccator for 60 min. Slides were then exposed in a Beta Imager (Biospace Lab) for 16 h. Surface radioactivity, expressed as cpm/mm^2^, was measured in the medial septum, lateral septum and olfactory tubercle for 5-HT transporter occupancy, and in the caudate-putamen and nucleus accumbens for 5-HT_1B_ receptor occupancy using Beta vision plus software (Biospace Lab). These regions were chosen *a priori* for receptor occupancy assays because they have strong and reliable specific binding signals for the radioligands relevant to the targets under study. Importantly, the fractional target occupancy estimates generated from these regions can be assumed to be representative of fractional occupancies for that same target throughout the brain, based on two concepts. First, once a drug passes the blood brain barrier (BBB), it reaches an equilibrium concentration in the biophase that is similar everywhere within the BBB. Second, the law of mass action dictates that fractional receptor occupancy depends only on the concentration of the drug in the biophase and its affinity for the target. Regional differences in the expression of a target are irrelevant for fractional occupancy measurements. Specific binding was determined by subtracting non-specific from total binding, and expressed as a percentage of mean specific binding in vehicle-treated control brains in the same assay. These values were subtracted from 100 to obtain fractional target occupancies.

### Statistical analyses

The investigator conducting the behavioral test was blind to the experimental treatment of the rat being tested. Trials to criterion (TTC) on the simple discrimination task on the training day were first compared by ANOVA to ensure that acquisition and general performance capability were comparable between experimental groups. Similarly, on the test day, performance on the simple and compound discriminations preceding the reversal learning task were analyzed to ensure that there were no group differences in the ability to acquire the contingencies. In experiment 1, the extent of 5-HT depletion was determined by comparing 5-HT concentration in the OFC of PCPA- and vehicle-treated rats using a *t*-test. TTC on the reversal learning task were compared between sub-chronic treatment groups using a two-way MANOVA (PCPA × sub-chronic vortioxetine). Acute vortioxetine treatment was compared to the vehicle control group in PCPA-pretreated rats by *t*-test. In experiment 2, two-way MANOVA (vortioxetine diet × CIC stress) was used to compare reversal learning between groups. Body weights and fractional target occupancies were analyzed by MANOVA. When significant main effects or interactions were detected, pair-wise comparisons were made using the Newman–Keuls *post-hoc* test.

## Results

### Experiment 1: effects of acute and sub-chronic vortioxetine treatment on reversal learning impairment induced by 5-HT depletion

**Fig. 3.**
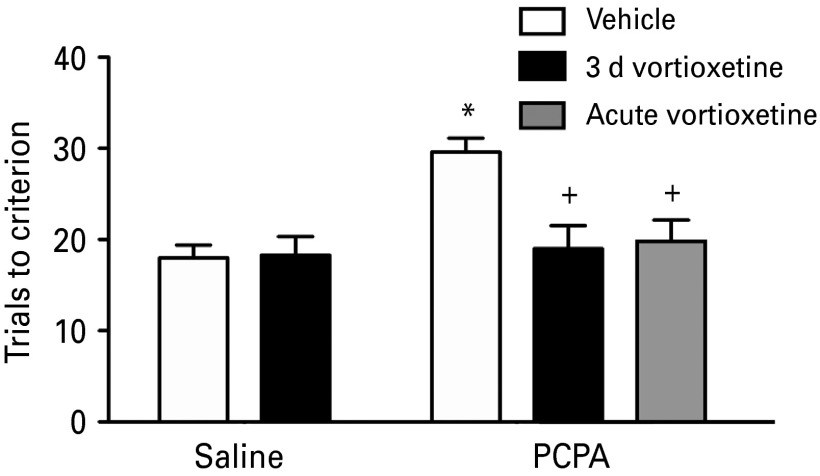
Effects of sub-chronic and acute vortioxetine treatment on reversal learning that had been compromised by serotonin depletion with 4-chloro-DL-phenylalanine methyl ester hydrochloride (PCPA). PCPA treatment significantly impaired reversal learning compared to saline pre-treated rats (*p < 0.01). Vortioxetine (10 mg/kg, i.p.) given once per day over 3 d, 30 min prior to habituation, training and testing on the attentional set-shifting test, significantly attenuated the reversal learning impairment in PCPA pre-treated rats compared to PCPA-pretreated rats given sub-chronic vehicle injections (+*p* < 0.001). A single acute injection of vortioxetine (10 mg/kg, i.p.) immediately before testing also significantly attenuated the PCPA-induced reversal learning impairment compared to vehicle treatment after PCPA (+*p* < 0.02). Data are shown as mean ± s.e.m (*n* = 7–8/group).

PCPA treatment significantly reduced 5-HT content in the OFC by 91.4 ± 1.2% (from 15.70 ± 2.80 to 1.47 ± 0.21 pg/mg; *t*_35_ = 6.16, *p* < 0.0001). All groups learned the simple discrimination during the training session comparably, indicating no pre-existing differences between treatment groups in their ability to acquire the contingencies and perform the required tasks as a result of PCPA (*F*_1,26_ = 1.60, *p* = 0.221) or vortioxetine (*F*_1,26_ = 0.293, *p* = 0.748) treatment. There were also no differences in performance on the two test stages prior to the reversal task of the AST (*p* = 0.905 and *p* = 0.830, Table 1). Figure 3 shows the effects of acute and 3 d sub-chronic vortioxetine treatment (10 mg/kg) in PCPA-pretreated rats. Replicating previous results, PCPA treatment impaired reversal learning, significantly increasing TTC on the reversal task compared to vehicle-treated controls (*F*_1,26_ = 9.70, *p* < 0.01). After sub-chronic treatment, there was a significant main effect of vortioxetine on reversal learning (*F*_1,26_ = 6.80, *p* < 0.02), and a significant PCPA × vortioxetine interaction (*F*_1,26_ = 7.58, *p* < 0.01). Subsequent *post-hoc* analyses showed that sub-chronic vortioxetine had no effect on reversal learning in control rats (*p* = 0.919), but significantly improved reversal learning in PCPA-pretreated rats (*p* < 0.001), reducing TTC on the reversal task to a level comparable to controls (Fig. 3). Similarly, single acute injection of vortioxetine immediately before testing also improved reversal learning in PCPA-treated rats (*t*_13_ = 2.80, *p* < 0.02 compared to vehicle injections, Fig. 3).

**Table 1.** Performance on the simple and compound discrimination tasks preceding the reversal learning task after PCPA and sub-chronic treatment with vortioxetine in Experiment 1

Group (Pretreatment, drug treatment)	Trials to criterion (mean ± s.e.m)	
	SD task	CD task
Saline, sub-chronic vehicle	11.4 ± 1.0	12.0 ± 2.2
Saline, sub-chronic vortioxetine (3 × 10 mg/kg)	10.7 ± 1.7	12.3 ± 2.4
PCPA, sub-chronic vehicle	13.6 ± 1.9	10.0 ± 2.1
PCPA, sub-chronic vortioxetine (3 × 10 mg/kg)	13.2 ± 1.0	9.4 ± 1.6

Data were analyzed by two-way ANOVA; all *p*-values >0.10.

There were no pre-existing group differences in starting body weight prior to PCPA treatment (*F*_1,26_ = 0.126, *p* = 0.725). Analysis of the percent change from baseline to final body weight (i.e. after 1 wk food restriction prior to testing) revealed no main effect of vortioxetine (*p* = 0.85) nor a PCPA × vortioxetine interaction (*p* = 0.749). There was a significant main effect of PCPA (*F*_1,26_ = 102.4, *p <* 0.001), with PCPA-treated rats showing a significantly greater loss of body weight than controls (saline–vehicle: −1.79 ± 0.74%; saline–vortioxetine: 1.92 ± 0.78%; PCPA–vehicle: −12.31 ± 1.38%; PCPA–vortioxetine: −11.63 ± 0.97%).

### Experiment 2: effects of chronic dietary administration of vortioxetine on reversal learning compromised by chronic intermittent cold (CIC) stress

Effects of dietary vortioxetine administration on 5-HT transporter and 5-HT_1B_ receptor occupancies in control and CIC stressed rats are presented in Table 2. Under *ad libitum* feeding conditions, there was a significant main effect of vortioxetine in the diet on both 5-HT transporter (*F*_2,15_ = 156.7, *p* < 0.0001) and 5-HT_1B_ receptor occupancy (*F*_2,15_ = 391.5, *p* < 0.0001). Chronic administration of vortioxetine at 0.6 g/kg of food led to 85–90% occupancy of the 5-HT transporter, and 55–60% occupancy of the 5-HT_1B_ receptor in control and CIC rats, respectively. The 1.8 g/kg vortioxetine diet led to full occupancy at both the 5-HT transporter and 5-HT_1B_ receptor targets. There was no effect of stress (5-HT transporter: *F*_1,15_ = 0.1, *p* = 0.752; 5-HT_1B_ receptor: *F*_1,15_ = 0.6, *p* = 0.443) nor a stress × vortioxetine interaction for either target (5-HT transporter: *F*_2,15_ = 0.1, *p* = 0.897; 5-HT_1B_ receptor: *F*_2,15_ = 1.2, *p* = 0.322).

**Table 2.** Rat brain 5-HT transporter and 5-HT_1B_ receptor occupancy (mean % ± s.e.m) after chronic treatment with vortioxetine in the diet

Vortioxetine concentration in the diet	5-HT transporter		5-HT_1B_ receptor	
	Control	CIC	Control	CIC^a^
*Ad libitum* feeding period				
Control diet	0 ± 17	0 ± 3	0 ± 0.5	0 ± 5
0.6 g/kg food^b^^,^^c^	86 ± 0.2	91 ± 1	54 ± 1.6	62 ± 5
1.8 g/kg food^b^^,^^c^	96 ± 0.5	96 ± 0.5	91 ± 1.6	90 ± 1
Period of food restriction (14 g food/day)				
Control diet	0 ± 4	0 ± 2	0 ± 6	0 ± 1
0.6 g/kg food^b^^,^^c^	48 ± 4	53 ± 10	33 ± 4	22 ± 9
1.8 g/kg food^b^^,^^c^	90 ± 2	82 ± 5	69 ± 3	36 ± 8

a 5-HT_1B_ receptor occupancy in CIC-stressed rats was significantly different from unstressed controls, in the restricted feeding condition only, *p* < 0.05.

b 5-HT transporter occupancy was significantly higher in vortioxetine-treated rats than in controls, *p* < 0.0001.

c 5-HT_1B_ receptor occupancy was significantly higher in vortioxetine-treated rats than in controls, *p* < 0.0001.

Under conditions of food restriction (14 g/d), for which the estimated daily drug dose was calculated, a significant main effect of vortioxetine in the diet was observed on occupancy at the 5-HT transporter (*F*_2,31_ = 58.5, *p* < 0.0001) and the 5-HT_1B_ receptor (*F*_2,31_ = 19.34, *p* < 0.0001). The 0.6 g/kg vortioxetine diet led to approximately 50% 5-HT transporter occupancy and 20–30% 5-HT_1B_ receptor occupancy in both control and CIC rats. The 1.8 g/kg vortioxetine diet led to full 5-HT transporter occupancy and 5-HT_1B_ receptor occupancy ranging from approximately 35% in CIC rats to 70% in control rats. Although there was no significant effect of stress on 5-HT transporter occupancy (*F*_1,31_ = 0.03, *p* = 0.859), there was a significant effect on 5-HT_1B_ receptor occupancy (*F*_1, 31_ = 5.1, *p* < 0.05), with CIC rats having significantly lower 5-HT_1B_ receptor occupancy than control rats. However, there was no significant stress × vortioxetine interaction at either target (5-HT transporter: *F*_2,31_ = 0.5, *p* = 0.608; 5-HT_1B_ receptor: *F*_2,31_ = 2.2, *p* = 0.125).

**Fig. 4.**
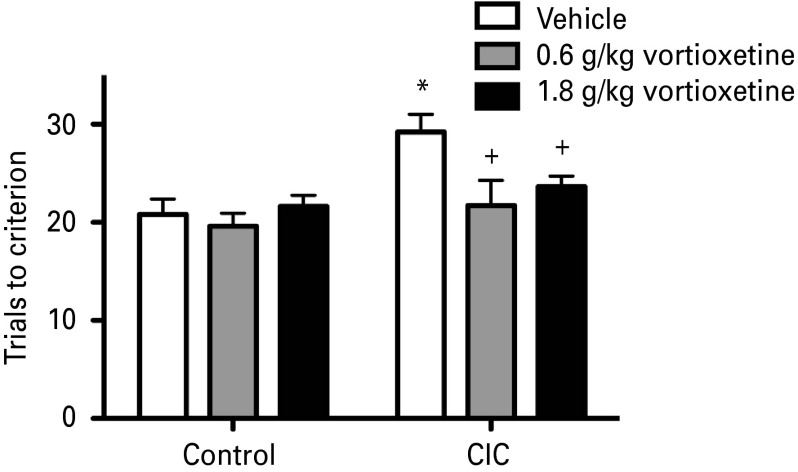
Effects of chronic dietary vortioxetine treatment, administered concurrently with chronic intermittent cold (CIC) stress, on reversal learning. CIC stress significantly impaired reversal learning (*p < 0.01, compared to vehicle-treated controls; mean ± s.e.m; *n* = 7–8 per group). Chronic treatment with both the low (30 mg/kg/day) and high dose (90 mg/kg d^−1^) of vortioxetine administered in the diet concurrently with CIC stress alleviated the stress-induced deficit in reversal learning (+*p* < 0.01 and *p* < 0.05, respectively, compared to vehicle-treated CIC-stressed rats; mean ± s.e.m; *n* = 8–11 per group).

Two-way ANOVA revealed no differences between groups in training (*F*_4,94_ = 1.92, *p* = 0.114) or in the test stages preceding the reversal learning task on the AST (*F*_4,94_ = 0.46, *p* = 0.767), indicating that all groups learned the contingencies and could perform the test comparably (Table 3). Figure 4 shows the effect of chronic dietary vortioxetine treatment (30 or 90 mg/kg d^−1^) on reversal learning in CIC-stressed rats. Two-way ANOVA indicated significant main effects of CIC stress (*F*_1,48_ = 8.32, *p* < 0.01), and chronic vortioxetine treatment (*F*_2,48_ = 3.24, *p* < 0.05). The interaction approached significance (*F*_2,48_ = 2.28, *p* = 0.11). *Post-hoc* analyses showed that CIC-stressed rats fed the control diet required significantly more trials to reach criterion on the reversal task than non-stressed rats fed the control diet (Fig. 4), replicating the detrimental effect of CIC stress on reversal learning reported previously (Lapiz-Bluhm et al., 2009). Chronic treatment with either dose of vortioxetine in the diet significantly attenuated the reversal learning impairment induced by CIC stress (*p* < 0.01 and *p* < 0.05, respectively, for low- and high-dose vortioxetine compared to control diet in CIC-stressed rats; Fig. 4).

**Table 3.** Performance on the simple and compound discrimination tasks preceding the reversal learning task after CIC stress and chronic dietary treatment with vortioxetine in Experiment 2

Group (Stress, Diet)	Trials to criterion (mean ± s.e.m)	
	SD Task	CD Task
No stress, Control diet	9.6 ± 0.9	10.5 ± 1.3
No stress, Low-vortioxetine diet (0.6 g/kg food)	11.4 ± 3.6	10.6 ± 1.0
No stress, High-vortioxetine diet (1.8 g/kg food)	11.8 ± 1.9	10.9 ± 1.8
CIC, Control diet	11.0 ± 1.4	13.9 ± 1.8
CIC, Low-vortioxetine diet (0.6 g/kg food)	10.5 ± 0.7	12.8 ± 2.8
CIC, High-vortioxetine diet (1.8 g/kg food)	11.6 ± 2.3	10.1 ± 1.3

Data were analyzed by two-way ANOVA; all *p*-values >0.54.

**Fig. 5.**
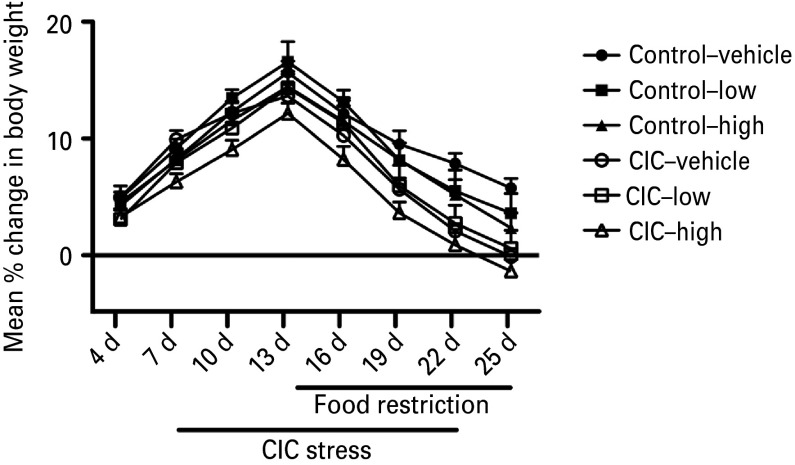
Body weight change over the course of chronic treatment with vortioxetine in the diet or control diet, including the 2 wk period of chronic intermittent cold (CIC) stress and the 10 d period of food restriction preceding behavioral testing (bars). The respective diets were maintained for one additional day after testing to obtain brain tissue for receptor occupancy analyses. Animals exposed to CIC stress showed less weight gain than unstressed controls (open symbols compared to filled symbols), and this difference reached statistical significance during the period of food restriction, during which all groups lost weight (significance at specific time points is not indicated for the sake of clarity). These effects were equivalent across the different dietary treatments, for which there were no main effects or interactions. Data shown as mean ± s.e.m (*n* = 8–11 per group).

There were no pre-existing differences in body weight between groups prior to treatment, with group means (±s.e.m) ranging from 281 ± 3 g to 293 ± 5 g. Analysis of body weight change over the course of the experiment included the 24 d dietary treatment with vortioxetine or control diet, the 2 wk period of CIC stress or unstressed control treatment, and the 10 d period of food restriction prior to testing (see Fig. 5). There were significant main effects of stress (*F*_1,47_ = 10.20, *p* < 0.01) and time (*F*_7,329_ = 245.51, *p* < 0.001), and a stress × time interaction (*F*_7,329_ = 6.79, *p* < 0.001). There were no main effects or interactions involving diet. All groups gained body weight equivalently during the 7 d free-feeding period preceding stress. There was a slight slowing of weight gain induced by CIC stress, which became significant, by *post-hoc* comparisons, only during the period of food restriction, during which all groups showed a decrease in body weight (see Fig. 5, with indicators of significance omitted for the sake of clarity). These patterns of effect on weight gain were equivalent across dietary treatments.

## Discussion

We investigated the effects of vortioxetine, a novel antidepressant drug with multimodal action, on impairments in reversal learning mediated in the OFC. To test the direct effects of vortioxetine acting at post-synaptic 5-HT receptors, we analyzed the effects of sub-chronic and acute vortioxetine administration on reversal learning impairments induced by 5-HT depletion with PCPA, effectively eliminating 5-HT reuptake inhibition as a potential mode of action. Secondly, we tested the efficacy of the full spectrum of pharmacological activity of vortioxetine in intact rats in which the reversal learning impairment was induced by exposure to a metabolic stressor, chronic intermittent cold stress, with vortioxetine administered chronically in the diet. The results of experiment 1 demonstrated a beneficial effect of vortioxetine, given acutely or sub-chronically, in alleviating the reversal learning impairment induced by PCPA, with no effect on cognitive performance in control rats. This suggests that the actions of vortioxetine at post-synaptic 5-HT receptors may contribute to its efficacy in improving cognitive flexibility. Further, chronic administration of vortioxetine in the diet reversed the CIC stress-induced deficit in reversal learning, suggesting that vortioxetine has beneficial effects in alleviating cognitive impairments in intact animals, in which its full spectrum of pharmacological effects on both pre- and post-synaptic 5-HT receptors as well as 5-HT reuptake were exerted.

Reversal learning is a form of cognitive flexibility, an executive process that allows the adaptive modification of behavior in response to changes in internal state or environmental circumstance (Rygula et al., 2010). The OFC is critical to this process, as lesions of the OFC result in perseveration and an inability to suppress previously reinforced responses in a reversal task in rodents and primates (Dias et al., 1996; McAlonan and Brown, 2003; Boulougouris et al., 2007). Studies of performance deficits in patients with focal cortical lesions, and neuroimaging studies in healthy volunteers have confirmed an important role for the OFC in reversal learning in humans (Fellows and Farah, 2003; Hampshire and Owen, 2006; Hampshire et al., 2012). Serotonin has been implicated in the modulation of reversal learning in the OFC (Clarke et al., 2007; Lapiz-Bluhm et al., 2009; Furr et al., 2012). We have shown previously that the deficit in reversal learning induced by CIC stress was reversed by chronic SSRI treatment (Lapiz-Bluhm and Morilak, 2010). In the present study, vortioxetine reversed the impairments induced by both CIC stress and 5-HT depletion. Vortioxetine is a multimodal-acting antidepressant; in addition to inhibiting 5-HT reuptake, it is an antagonist at 5-HT_3A_, 5-HT_7_ and 5-HT_1D_ receptors, a partial agonist at 5-HT_1B_ receptors, and a full agonist at 5-HT_1A_ receptors (Bang-Anderson et al., 2011). Thus, given its multitude of pharmacological actions on serotonergic transmission, the beneficial effects of vortioxetine on reversal learning could be mediated by a combination of 5-HT reuptake inhibition elevating extracellular 5-HT levels together with direct activity at specific 5-HT receptors.

The beneficial effect of acute vortioxetine administration on the reversal learning impairment induced by 5-HT depletion is likely due to direct post-synaptic 5-HT receptor agonist activity, namely at 5-HT_1A_ and/or 5-HT_1B_ receptors. In a previous study, acute administration of 10 mg/kg vortioxetine to PCPA-treated rats lead to functionally relevant occupancies of 5-HT_1A_ and 5-HT_1B_ receptors (43 and 89%, respectively), and recovery of memory impairment in the object recognition and spontaneous alternation tests (du Jardin et al., 2014). Autoradiographic studies have shown a high density of 5-HT_1A_ and 5-HT_1B_ receptors located on post-synaptic neurons in limbic forebrain areas such as the hippocampus, lateral septum and prefrontal cortex, where they may influence cognitive and emotional processes (Pazos and Palacios, 1985; Lucki et al., 1994). Activation of 5-HT_1A_ receptors decreased immobility on the rat forced swim test (FST) and this was blocked by pretreatment with 5-HT_1A_-receptor antagonists (Detke et al., 1995; Cryan et al., 2005). When serotonergic neurons were selectively destroyed or 5-HT synthesis inhibited, 5-HT_1A_ receptor agonists still produced antidepressant-like effects in the FST, suggesting that these effects were due to direct activation of postsynaptic 5-HT_1A_ receptors (Lucki et al., 1994). Similarly, the 5-HT_1B_-receptor agonists, anpirtoline and CP94253, also produced antidepressant-like effects in the FST in mice (Tatarczynska et al., 2005; Chenu et al., 2008). These effects were blocked by antagonism or genetic deletion of the 5-HT_1B_ receptor, but not by selective lesions of 5-HT neurons, providing further evidence for a post-synaptic 5-HT_1B_ receptor-mediated effect (Chenu et al., 2008). Moreover, 5-HT_1B_ receptor antagonists blocked the decrease in immobility produced by citalopram, suggesting that 5-HT_1B_ receptors may contribute to the antidepressant effects of SSRIs (Chenu et al., 2008).

Some of the effects of vortioxetine on reversal learning after 5-HT depletion could have been mediated by compensatory responses in other monoaminergic systems, specifically the noradrenergic system, which facilitates extradimensional set-shifting, another component of cognitive flexibility mediated in the medial prefrontal cortex (mPFC) (Lapiz and Morilak, 2006). Although vortioxetine has low affinity for adrenergic receptors and the NE transporter (Bang-Anderson et al., 2011), increases in extracellular NE levels in the mPFC have been demonstrated following acute administration of vortioxetine, whereas the selective SSRI, escitalopram, had no effect (Pehrson et al., 2013). In other microdialysis experiments, elevated NE levels were observed in the cortex, hippocampus and hypothalamus after systemic administration of 5-HT_1A_ receptor agonists (Suzuki et al., 1995; Suwabe et al., 2000). Thus, vortioxetine acting as an agonist at post-synaptic 5-HT_1A_ receptors may increase NE transmission. Elevating NE acutely by administration of the *α*_2_-adrenergic autoreceptor antagonist, atipamezole, to naïve rats facilitated both reversal learning and extradimensional set-shifting on the AST (Lapiz and Morilak, 2006). However, elevation of NE alone is unlikely to account for the beneficial effects of vortioxetine on reversal learning in the present experiments, as chronic treatment with desipramine, a selective NE reuptake inhibitor, failed to alleviate the CIC stress-induced impairment in reversal learning (Lapiz-Bluhm and Morilak, 2010).

Both the OFC and mPFC receive serotonergic innervation from the dorsal raphe nucleus (DRN), and a reciprocal projection from the PFC to the DRN provides a neural substrate for top-down control of activity in forebrain-projecting serotonergic pathways (Amat et al., 2005). Stimulation of pre-synaptic 5-HT_1A_ and 5-HT_1B_ autoreceptors inhibits 5-HT neuronal firing, synthesis and terminal release, and it has been proposed that autoreceptor desensitization may be responsible for the delay between initiating antidepressant treatment and reduction of depressive symptoms (Piñeyro and Blier, 1999; Artigas et al., 2001). Similarly, long term treatment with 5HT_1A_ receptor agonists desensitized 5-HT_1A_ autoreceptors but not post-synaptic 5-HT_1A_ receptors on CA3 pyramidal neurons (Blier and de Montigny, 1987). Rapid desensitization of somatodendritic 5-HT_1A_ receptors has been reported after 1–3 d of vortioxetine treatment, whereas 14 d of treatment with fluoxetine were required to achieve this same effect (Bétry et al., 2013). Thus, activation of 5-HT_1A_ receptors by vortioxetine may expedite a therapeutic response by rapidly desensitizing autoreceptors, further increasing 5-HT release while also blocking reuptake, which then activates post-synaptic 5-HT receptors that facilitate reversal learning. However, it is important to emphasize that while desensitization of 5-HT autoreceptors may contribute to the efficacy of vortioxetine in intact animals, it is unlikely to have played a role in the beneficial effects of sub-chronic or acute vortioxetine administration in restoring reversal learning in rats compromised by 5-HT depletion.

In intact animals exposed to CIC stress, chronic treatment with the SSRI, citalopram, reversed the deficit in cognitive flexibility, suggesting that elevating 5-HT transmission in the OFC is sufficient for effects on reversal learning (Lapiz-Bluhm et al., 2009; Lapiz-Bluhm and Morilak, 2010). Vortioxetine was shown to increase 5-HT to an even greater extent than escitalopram (Pehrson et al., 2013), indicating that when the 5-HT receptor actions of vortioxetine are exerted in combination with reuptake inhibition, a greater elevation in extracellular 5-HT levels is attainable than with reuptake blockade alone. In the present study, the low dose of vortioxetine, corresponding to approximately 50% occupancy of the 5-HT transporter, fully restored the stress-induced impairment of reversal learning. SSRIs generally require higher levels of 5-HT transporter occupancy to be active in both preclinical and clinical contexts (Meyer, 2007; Kreilgaard et al., 2008). Occupancy of the 5-HT transporter at clinically approved doses of vortioxetine is approximately 50% at 5 mg, 65% at 10 mg, and >80% at 20 mg (Areberg et al., 2012; Stenkrona et al., 2013). In rodents, extracellular 5-HT was increased at transporter occupancy as low as 40% (Mørk et al., 2012).

It seems at first unlikely that the 5-HT receptor antagonist properties of vortioxetine could account for its effects on reversal learning in PCPA-treated rats, in which there was >90% depletion of 5-HT in the OFC. However, in a previous study, acute vortioxetine administration restored PCPA-induced memory deficits at a lower dose than in the present study (0.1 mg/kg), at which only 5-HT_3_ receptors and the 5-HT transporter were occupied, and so did the 5-HT_3_ receptor antagonist, ondansetron (du Jardin et al., 2014). Thus, it is possible that 5-HT_3_ antagonist activity could have had a role in acutely restoring the PCPA-induced reversal learning deficit in our study as well. More importantly, this observation suggests that blockade of 5-HT_3_ receptors may have contributed to attenuation of the CIC stress-induced reversal learning deficit by chronic dietary administration of vortioxetine in intact rats. The 5-HT_3_ receptor is found primarily on cortical and hippocampal GABAergic interneurons (Morales et al., 1996; Puig et al., 2004). Blockade of this receptor potentiates the increase in extracellular 5-HT produced by citalopram (Mørk et al., 2012) and augments the effects of sub-threshold doses of SSRIs in the mouse FST (Ramamoorthy et al., 2008). Thus, 5-HT_3_-receptor antagonism by vortioxetine may enhance the elevation in extracellular 5-HT levels produced by reuptake blockade, perhaps by reducing activity of GABAergic interneurons and disinhibiting serotonergic transmission in the OFC, contributing to the facilitation of reversal learning.

Another possible antagonist target of vortioxetine is the 5-HT_7_ receptor, expressed mainly in the thalamus, hypothalamus, hippocampus and cortex (Hedlund and Sutcliffe, 2004). Blockade of 5-HT_7_ receptors has antidepressant-like effects in the mouse FST and tail suspension tests (Sarkisyan et al., 2010), and a synergistic interaction was seen between sub-effective doses of 5-HT_7_ receptor antagonists and SSRIs on the same tests (Bonaventure et al., 2007). Further, selective 5-HT_7_ receptor antagonism facilitated extradimensional set-shifting in chronically-stressed and unstressed control rats and promoted cognitive flexibility given in combination with a sub-effective dose of escitalopram in naïve rats (Nikiforuk, 2012). Thus, 5-HT_7_ receptor blockade may also contribute to the effectiveness of vortioxetine in restoring reversal learning compromised by chronic stress.

In reversal learning, animals must inhibit previously learned or pre-potent responses to acquire a new response and adapt to a change in the environment, e.g. a new reward contingency. Reductions in 5-HT neurotransmission in the OFC by either chronic stress or 5-HT depletion disrupt this ability (Lapiz-Bluhm et al., 2009; Lapiz-Bluhm and Morilak, 2010). In humans, cognitive flexibility deficits may contribute to perseverative emotional biases involved in the development and maintenance of depressive symptoms (Beck, 1976; Mathews and Mackintosh, 1998; Coles and Heimberg, 2002). Cognitive deficits and compromised executive function often persist as residual symptoms after antidepressant treatment, and may limit the extent of recovery and predispose patients to relapse (Eaves and Rush, 1984; Jaeger et al., 2006; Herrera-Guzmán et al., 2010). A significant advance represented in the current results is the possibility that the multimodal pharmacological properties of vortioxetine may offer additional mechanisms beyond serotonin reuptake blockade for the management of cognitive deficits in depression, which may improve treatment outcome. Additional clinical studies are required to investigate this possibility further.

## Statement of Interest

Other than the receipt of research funding from Lundbeck to support this work, A. Wallace and D. Morilak have no other interests to disclose. A. Pehrson, and C. Sánchez are Lundbeck employees.
